# Investigating the role of tripartite motif containing-21 and interleukin-6 in pro-Inflammatory symptom-associated heterogeneity within primary Sjögren’s syndrome

**DOI:** 10.1515/rir-2025-0019

**Published:** 2025-10-04

**Authors:** Lauren R Scott, Joe Berry, Kyle Thompson, Jessica Tarn, Karl Wood, John Casement, Wan-Fai Ng

**Affiliations:** Translational and Clinical Research Institute, Newcastle University, Newcastle upon Tyne, UK

**Keywords:** Sjögren’s Syndrome, rheumatology, inflammation

## Abstract

**Background and Objectives:**

In Primary Sjögren’s Syndrome (pSS) clinical heterogeneity is a challenge to both treatment and disease understanding. Variations in symptoms may be driven by different underlying biological pathways. Tripartite motif containing-21 (TRIM21) and interleukin-6 (IL-6) have been implicated in autoimmunity and inflammation, with links to chronic interferon activity. We assess the levels of TRIM21 and IL-6 in the context of anti-Ro autoantibody status, and in different symptom-based pSS subgroups we have previously described, to explore whether they may contribute to the clinical heterogeneity in pSS.

**Methods:**

We measured serum IL-6 concentrations for 193 pSS patients and 18 healthy controls, and analysed available microarray data for TRIM21 transcript expression in 184 pSS patients and 33 healthy controls. Levels of IL-6 and TRIM21 were analysed in the context of symptom-based subgroups, anti-Ro autoantibody status and symptom scores.

**Results:**

TRIM21 and IL-6 levels were significantly raised in pSS patients compared to healthy controls. TRIM21 expression was similar between symptom-based subgroups, whilst IL-6 concentrations were significantly increased in the high symptom burden group compared to the low symptom burden group. TRIM21 levels were significantly increased in Ro+ autoantibody groups compared to Ro-, whilst IL-6 levels were similar between groups.

**Conclusions:**

Our results suggest a potential role for IL-6 in the pathogenesis of the high symptom burden group. Increased TRIM21 transcript levels in the Ro+ group supports the hypothesis of suggests autoantibody targeting of the TRIM21 protein leading to aberrant type I interferon (IFN) production which in turn may drive further TRIM21 transcript production.

## Introduction

Primary Sjögren’s Syndrome (pSS) is a chronic autoimmune disease, primarily affecting the salivary and lacrimal glands, however systemic manifestations can occur. Symptoms vary in severity, and can include: fatigue, joint pain, debilitating dryness particularly to the eyes and mouth, depression and anxiety.^[1]^ Despite the fact that symptoms can vary in both severity and constitution, Health-related quality of life (HR-QoL) measures are significantly reduced for these patients irrespective of their symptom presentation, with longitudinal data from the Newcastle pSS cohort demonstrating a substantial decline in HR-QoL, which positively correlated to disease burden. Given the association between symptom measures and quality of life, it is likely that symptomatic improvement in pSS would lead to improved HR-QoL.^[2]^

On the basis of the aforementioned variations in both symptom severity ant type, there is recognised heterogeneity in pSS patients, which highlights its complexity and accounts, in part, to the fact that there is no cure, with symptomatic improvement being the key, unmet need for patients.^[1]^ Previous work in our group therefore focused the clustering of this highly heterogenous patient population into subgroups on the basis of symptom prevalence. We previously described four symptom-based subgroups of pSS; Low symptom burden (LSB), High symptom burden (HSB), Pain dominant with fatigue (PDF) and Dryness dominant with fatigue (DDF), with different transcriptomic and serum proteomic profiles which may responds to different therapies.^[3,4]^ Further elucidation of biological differences between these subgroups may contribute to greater understanding of the underpinning biological basis of symptom heterogeneity and better therapeutic targeting.^[4,5]^

Chronic inflammation is a key feature of autoimmunity, and therefore variations within the biological mechanisms underpinning this inflammation may contribute to variations within these patients. Persistent activation of pro-inflammatory cytokine and interferon (IFN) systems is recognised in pSS but not yet fully understood within pSS clinical heterogeneity, with no further investigation into the associations between this mechanistic activation and the manifestations of certain symptom combinations within the previously established symptom-based subgroups.^[6, 7, 8, 9]^ Tripartite motif containing-21 (TRIM21) is a major autoantigen in pSS.^[10]^ It functions as an E3 ubiquitin ligase which is present in haematopoietic cells and can ubiquitinate IFN regulatory factors (IRFs), which in turn may regulate the type I IFN response, thereby linking the concepts of IFN activation and autoantigen in pSS. TRIM21 has been shown to stabilise or degrade IRFs, suggesting a regulatory role in maintaining IFN signalling balance and preventing exaggerated type I IFN response.^[11,12]^ Furthermore, activation of type I IFN system in pSS patients is associated with the presence of anti-Ro autoantibody. It is plausible that TRIM21 expression levels may contribute to IFN-associated clinical heterogeneity.^[13]^

Whilst the investigation into IFN may elucidate previously unknown associations between patient groups, serum-based cytokines have also been shown to vary significantly in pSS patients and therefore investigation into this may also contribute to the increasing knowledge of biological variation.^[4]^ Interleukin-6 (IL-6) plays key roles in chronic inflammation.^[14]^ Hulkkonen *et al*. demonstrated that plasma IL-6 levels were significantly greater in pSS patients compared to healthy controls, and that IL-6 level increased in parallel to the histological grade of minor salivary gland biopsy and to number of pSS criteria met.^[15]^ Furthermore, Melo *et al*. demonstrated that higher serum levels of IL-6 correlate with greater disease activity in pSS patients.^[16]^ However, whether IL-6 may also contribute to symptom heterogeneity in pSS has not been explored. We aim to investigate the relationship between TRIM21 and IL-6 expression within different symptom-based subgroups of pSS patients, in order to explore their roles, if any, in the symptom heterogeneity among pSS patients.

## Materials and Methods

### Patients

Serum samples of 193 pSS patients were taken from the United Kingdom Primary Sjögren’s Syndrome Registry (UKPSSR) biobank; which includes biological samples from over 1200 patients with clinically well-characterised pSS.^[17]^

All patients had given written consent according to the principles of the Helsinki Declaration, and ethical approval for this study was granted by the UK National Research Ethics Committee (North West-Haydock).

### ELISA

Serum IL-6 levels were measured using enzyme-linked immunosorbnent assay (ELISA, Invitrogen Cat: KHC0061) according to the manufacturer’s protocol. Samples were selected to be similarly distributed between the symptom-based subgroups.

### Microarray

Microarray data for TRIM21 transcript expression for 184 pSS patients and 33 healthy controls was obtained from our previous work. Microarray data assessed the level of TRIM21 transcripts from peripheral blood of patients.

### Clinical Measures

IL-6 and TRIM21 data was analysed against symptom-based subgroups and individual symptoms; fatigue, pain, dryness, anxiety, depression,^[18]^ which was collected at the same time as sample collection, and autoantibody status, obtained from patient medical records.

### Statistical Analysis

All statistical analysis was undertaken using Prism (GraphPad) V.9. Kruskal-Wallis (non-parametric ANOVA) and Man-Whitney (non-parametric t-test) were used to analyse differences between groups. For correlation analysis, Spearman R correlation testing was used (non-parametric). For all tests, statistical significance was defined as *P* < 0.05.

## Results

As previously mentioned, the patient cohort consisted of 184 patients for the microarray analysis and 193 patients for the IL-6 analysis. These cohorts differed however, so the analysis was not paired. Both cohorts of patients were made up of patients from the UKPSSR biobank; well-characterised patients. Table 1, below, demonstrates the detailed demographics for both these cohorts.

**Table 1:** The demographics of the patients for the two experimental cohorts of TRIM21 microarray analysis and IL-6 ELISA analysis.

Parameters	Cohort	
	TRIM21 (*n* = 184)	IL-6 (*n* = 193)
AGE		
Mean (years)	57.5	60.3
SEX		
Female (%)	92.9	95.9
Male (%)	7.1	4.1
ETHNICITY		
Caucasian (%)	91.9	94.3
African (%)	1.6	2.1
Asian (%)	3.3	2.6
Other (%)	3.2	1.0
SYMPTOM-SUBGROUP		
Low Symptom Burden (LSB) (%)	30.98	27.46
High Symptom Burden (HSB) (%)	27.17	25.38
Pain Dominant with Fatigue (PDF) (%)	29.35	23.32
Dryness Dominant with Fatigue (DDF) (%)	12.50	23.83
ANTIBODY STATUS		
Anti-Ro Positive (%)	86.96	84.97
Anti-Ro Negative (%)	13.04	15.03

Cohorts of both patients are displayed side-by-side to demonstrate cohort similarity and non-bias. Demographics are shown for subheadings of age, sex, ethnicity, symptom-subgroup and antibody status. TRIM21, tripartite motif containing-21; IL-6, interleukin 6; ELISA, enzyme-linked immunosorbnent assay.

As shown in the above table, demographics for the TRIM21 microarray and the IL-6 ELISA cohort represented consistent and similar demographics and clinical characteristics at the point of sample acquisition. The mean ranges are comparable along with sex and ethnicity. It should be noted at this point that the skew of demographics in both cohorts towards Caucasian females is representative of the normal disease demographics seen in a natural population.

Additionally, the antibody status, which is assessed in this paper is comparable between cohorts and as with the sex and ethnicity characteristics, the skew to anti-Ro positive status is also representative of natural disease trends, with seropositive disease being much more common than seronegative.

The clinically well-characterised nature of the two cohorts allows comparison of markers at a population and symptom-subgroup trend level despite the fact that data are not paired.

### PSS vs. Healthy Controls

**Figure 1:**
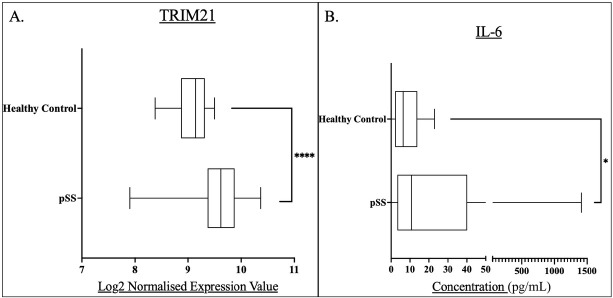
Box and whisker plots and associated descriptive statistics tables demonstrating the level of: A) TRIM21 expression in pSS patients (n = 184) compared to healthy controls (n = 33). B) IL-6 concentrations in pSS patient serum (n=193) compared to that of healthy controls (n = 18). Levels of significance were defined as P<0.05. Levels of significance are shown by Asterix number: *P<0.05, ****P<0.0001. Graphs were generated in GraphPad prism V. 9.

Initial analysis demonstrated significantly higher TRIM21 and IL-6 levels among pSS patients compared to healthy controls (Figure 1).

Both TRIM21 and IL-6 expression were significantly higher in pSS patients compared to healthy controls, suggesting a role for these proteins in pSS pathogenesis. TRIM21 levels were significantly greater in all symptom-based subgroups compared to healthy controls, however there was no difference between these subgroups. IL-6 concentration was significantly higher in the HSB subgroup compared to the LSB subgroup (Table 1).

The pSS group represented a median TRIM21 expression of 9.616, greater than the healthy controls with an expression of 9.141, The pSS group expression was shown to be significantly greater than that of the healthy controls, (*P* < 0.0001).

The pSS group demonstrated a greater median IL-6 concentration compared to healthy controls at 10.76 ng/L versus 6.431 ng/L, this concentration difference was shown to be statistically significant, (*P* = 0.0485).

This analysis served the basis of further investigation, as there was clear indication of the role of both of these markers within pSS.

### Symptom-based Subgroups

**Figure 2:**
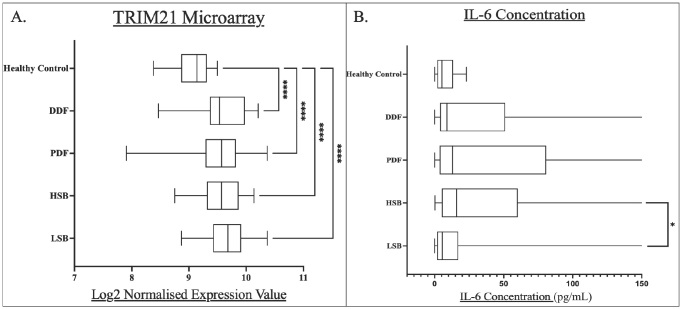
Box and whisker plots for each pSS subgroup compared to healthy controls for A) whole blood TRIM21 transcript levels, and B) serum IL-6 concentrations. Levels of statistical significance were denoted as follows: P<0.05. Levels of significance are shown by Asterix number: *P<0.05, ****P<0.0001. Median values were shown within the box plots.

Given previous research from this group focusing on the sub-classification of pSS into the symptom-based subgroups, it is of interest whether the expression of these markers differed between patients experiencing certain symptom prevalence. Figure 2 below demonstrates the analysis of these markers between subgroups and all individually compared to healthy controls as hope to elucidate symptom-related biological differences.

TRIM21 levels were similar between all pSS subgroups. All symptom-based subgroups had a significantly higher TRIM21 expression compared to healthy controls (*P* < 0.0001)(Figure 2A).

The HSB subgroup had the highest median serum level of IL-6 (15.88 ng/L) while the LSB subgroup had the lowest level (5.56 ng/L). The difference between HSB and LSB subgroups was statistically significant (*P* = 0.0024).

The HSB subgroup is characterised by high scores across all symptoms. We have previously demonstrated serum IL-6 levels to be highest in the HSB groups using OLINK technology.^[4]^ This is further validated by increased concentrations in ELISA.

### Relationship with pSS Symptoms

Weak but significant correlations were present between total symptom burden scores and TRIM21 (*R* =-0.19, *P* = 0.01) and IL-6 (*R* = 0.16, *P* = 0.03) (Supplementary Figure 1).

**Figure 3:**
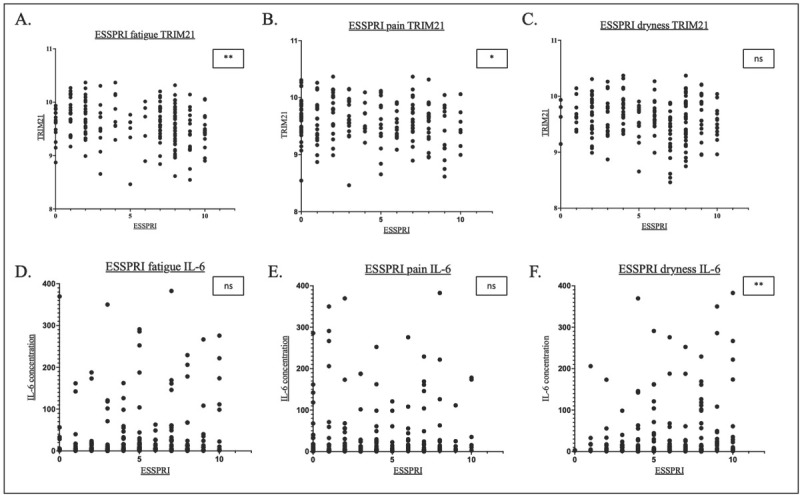
Scatterplots showing the correlation between fatigue, pain and dryness respectively and TRIM21 (A-C) and IL-6 (D-F). *P<0.05, **P<0.01. ns, not significant.

The correlation between TRIM21 and IL-6 levels and individual pSS symptom scores (ESSPRI) is shown in Figure 3.

TRIM21 expression was negatively correlated with fatigue (*R* = -0.22, *P* < 0.01) and pain (*R* =-0.16, *P* = 0.003)(Figure 3A-C). There was a trend of inverse relationship between TRIM21 expression and symptoms of dryness (*R* =-0.12, *P* = 0.11).

In contrast, serum IL-6 levels were weakly positively correlated with symptoms of dryness (*R* = 0.18, *P* = 0.02). There were trends of weak positive correlations between serum IL-6 and fatigue (*R* = 0.12, *P* = 0.11) and pain (*R* = 0.11, *P* = 0.12) (Figure 3D-F).

Since serum IL-6 level was highest in the HSB group, which is characterised by high levels of anxiety and depression, we also analysed the correlation between serum IL-6 level and anxiety and depression scores (Supplementary Figure 2). We found that anxiety scores were positively correlated with serum IL-6 levels (*R* = 0.16, *P* = 0.02).

With regard to symptoms, both ESSPRI fatigue and pain significantly correlated to increased levels of TRIM21 whilst only ESSPRI dryness significantly correlated to IL-6 concentrations (Figure 3A, B, F, Supplementary Figure 6A).

### Anti-Ro Autoantibody Status

**Figure 4:**
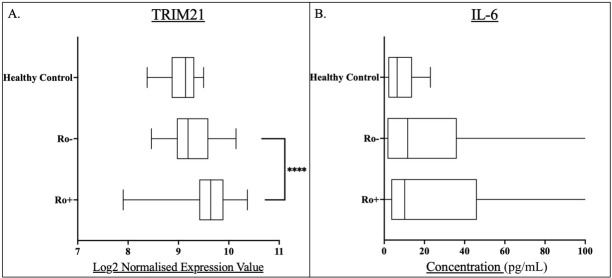
Box and whisker plots for anti-Ro positive (Ro+) and anti-Ro negative (Ro-) pSS patients and compared to healthy controls for A) whole blood TRIM21 transcript levels, and B) serum IL-6 concentrations. Levels of statistical significance were denoted as follows: P< 0.05. Levels of significance are shown by Asterix number: ****P<0.0001. Median values were shown within the box plots.

The median normalised expression value of TRIM21 in anti-Ro+ patients (9.64) was significantly higher compared to Anti-Ro patients (9.19)(*P* < 0.0001), which was similar to that of the healthy controls (*P* = 0.17) (Figure 4A). Inclusion of anti-La autoantibody in the stratification showed that TRIM21 expression was highest in Ro+/La+ patients (Supplementary Figure 3).

The IL-6 concentrations of the Anti-Ro+ and Anti-Ro- groups were comparable (*P* = 0.39)(Figure 4B).

TRIM21 expression was significantly increased in the Ro+ group compared to the Ro-, whilst no differences in IL-6 concentration was seen in relation to Ro+/Ro- groupings. (Figure 2) Further analysis demonstrated that the Ro+/La+ group had higher TRIM21 expression than Ro+/La- patients (Supplementary Figure 7).

## Discussion

The analysis of IL-6 is of interest given the known inflammatory effects of IL-6 in autoimmunity along with the relevance to IFN *via* STAT signalling pathways.

Studies have supported increased levels of IL-6 in pSS. Yoshimoto *et al*. demonstrated that compared to healthy controls, pSS PBMCs produce more IL-6 with or without stimulation with interferon gamma (IFN-γ).^[19]^

We demonstrate that TRIM21 transcript levels significantly correlate to symptoms of fatigue. Further investigation into this, however, is required and elucidation of either a direct or indirect link between TRIM21 and fatigue is yet to be established.

IL-6 has been implicated in depression and fatigue with evidence that increased IL-6 concentrations have been linked to depressive symptoms and increased circulating IL-6 linking to fatigue.^[4,20]^

IL-6 concentration has been implicated in altered stress responses. Increased IL-6 activity may lead to depression *via* Hypothalamic-Pituitary-Adrenal (HPA) axis activation.^[21]^ Additionally HPA Axis changes have been linked to chronic fatigue.^[22]^ Additional evidence from IL-6 knockout mice has linked depressive behaviours to the presence of this cytokine.^[23]^

In the present study however, we did not observe significant relationships between symptoms of depression and IL-6 concentration. This could be down to sample size. Given the natural variations in serum cytokine levels across patients, it may be that larger cohorts are required to see this trend. This may also be relating to the possibility that some patients may have symptoms of depression that are independent of their pSS. However, we found weak correlations between anxiety and IL-6 concentrations which could support the potential psychological symptom link with IL-6 given that anxiety and depression often coexist and may have overlapping aetiology.

We demonstrate that IL-6 levels significantly correlate with symptoms of dryness (Figure 1F). IL-6 levels are increased in both serum and saliva of pSS patients.^[24]^

Nandula *et al*. demonstrated that innate immune activation by Tol-like-receptor-3 agonist, poly (I: C) lead to a loss of salivary gland function in mice. This loss was not autoantibody mediated and fully reversible by removal of the innate immune stimulus. The stimulation caused an elevation of Type I IFN and IL-6 levels in the gland and systemically. In IL-6^-/-^ mice this loss of function was drastically reduced, highlighting the role of increased IL-6 in this salivary gland dysfunction. Whilst the exact mechanism by which this occurs remains unknown, the authors hypothesise IL-6 and Type I IFN mediated interference with the intracellular Ca^2+^ saliva production pathways.^[25]^

This theory would support our observations in this paper for the link between IL-6 and dryness.

Interestingly, Bettacchioli *et al*. found that Ro/La+ patients had a significantly increased disease activity score and significantly increased Type I and II IFN signature scores compared to both Ro+/La- and Ro-/La- groups.^[26]^

TRIM21 normally inhibits Type I IFN responses via Interferon regulatory factor (IRF) ubiquitination and additionally, TRIM21 is inducible by IFN highlighting the presence of TRIM21 in the IFN system.^[27]^

Kunishita *et al*. demonstrated that serum levels of Anti-Ro positively correlated to levels of serum Type I IFN. These same patients demonstrated reduced TRIM21 proteins expression in PBMCs, suggesting targeting and degradation of the TRIM21 by targeting with the autoantibody, leading to reduced capacity for IFN inhibition and regulation.^[28]^

Additionally, Kamiyama *et al*. demonstrated that in healthy controls elevated IFN transcripts associate with decreased TRIM21 mRNA. Interestingly, in anti-Ro positive systemic lupus erythematosus (SLE) patients, increased TRIM21 mRNA associated to increased Type I IFN. There was no correlation between IFN and TRIM21 transcripts in the Anti-TRIM21 negative patients suggesting a role of the autoantibody in TRIM21 dysregulation observed.^[29]^

In this paper we show that significantly increased TRIM21 transcript is present in Ro+ patients compared to Ro- patients, supporting the evidence in the previously discussed literature.

It could therefore be hypothesised that in pSS, the normal regulation of Type I IFN is disrupted due to the Anti-TRIM21 autoantibody. This autoantibody could be targeting the TRIM21 protein for degradation and therefore eliminate the ability to inhibit further IFN production. This could lead to the persistent exacerbated IFN signature seen in pSS patients.

In turn, the increased IFN may produce more TRIM21 mRNA, leading to a potential feedback loop of autoantibody mediated degradation and loss of IFN regulation, however the mechanism behind this remains unclear and further investigation into this hypothesis, and the mechanisms, is required.

## Limitations

One limitation of this study is that there are no available demographics for the healthy controls. There may be cases where variable such as age may infringe on pSS versus Healthy control results, however due to the nature of the collection these were anonymised. The pSS subgroups themselves however represent similar average ages and therefore the differences seen here are age-independent. Other limitations include the relatively small sample size and the presence of only UK based patients, therefore requiring validation in independent cohorts.

A further limitation of our study is the fact that we did not have a paired cohort for our TRIM21 analysis and our IL-6 analysis. This is due to access to samples and the need to have large cohort sizes for intra-subgroup analysis. Whilst this does not allow direct clinical comparison at the patient level this allows analysis at a patient cohort level which we believe to be of high interest.

Additionally, we were unable to test our hypothesis *via* mechanistic studies, at this stage our hypothesis adds to a growing body of evidence of this association and further studies aim to assess this.

Further analysis of this work with direct correlation with IFN scores would be of great interest and at present, ongoing work with collaborators look to investigate this fully.

## Conclusions

In conclusion, tis work demonstrates a promising link between the roles of both TRIM21 and IL-6 in relation to symptoms experienced by patients with pSS. This research forms good basis for further mechanistic validation, paired analyses and longitudinal studies within this patient to further investigate these trends which may be beneficial in elucidating pathways which influence the inflammatory state in these patients.

## Supplementary Material

Supplementary Material Details
