# Supplementary material for: Investigating the role of tripartite motif containing-21 and interleukin-6 in pro-Inflammatory symptom-associated heterogeneity within primary Sjögren’s syndrome
Source: Rheumatol Immunol Res. 2025 Oct 4;6(3):159–67. doi: 10.1515/rir-2025-0019 (PMC12495988; doi:10.1515/rir-2025-0019)
Supplement: Supplementary file 1 — Supplementary Material Details [file rir-2025-0019_sm.pdf]

SUPPLEMENTAL MATERIALS

Reevaluating Risk Assessment in CTD-PAH: The Prognostic Superiority of Stroke Volume Index

Improved CTD-PAH Risk Prediction Using Stroke Volume Index

Cai Qingqing[1,2,*], Ye Huangshu[2,*], Zhang Yixin[3,*], Dai Jiayi[3], Shan Linwei[3], Zhou Zhangdi[2], Li Dongyu[2], Liu Ting[4], Zhou Yanli[3], Yuan Fenghong[4,#], Sun Xiaoxuan[2,#]

[1]Department of Gerontology，Nanjing Hospital of Chinese Medicine Affiliated to Nanjing University of Chinese Medicine, China

[2]The Department of Rheumatology, The First Affiliated Hospital with Nanjing Medical University, Nanjing, China

[3]The Department of Cardiology, The First Affiliated Hospital with Nanjing Medical University, Nanjing, China

[4]The Affiliated Wuxi People's Hospital of Nanjing Medical University, Wuxi, China

*Cai Qingqing, Ye Huangshu and Zhang Yixin contributed equally to this work.

[#]**Corresponding author**:

Dr. Sun Xiaoxuan (main), The First Affiliated Hospital with Nanjing Medical University, No. 300 Guangzhou Road, Nanjing, Jiangsu Province 210029, China. Email: drsunxiaoxuan@163.com

Dr. Yuan Fenghong, The Affiliated Wuxi People's Hospital of Nanjing Medical University, Wuxi, China. Email: Yfh222222@163.com.

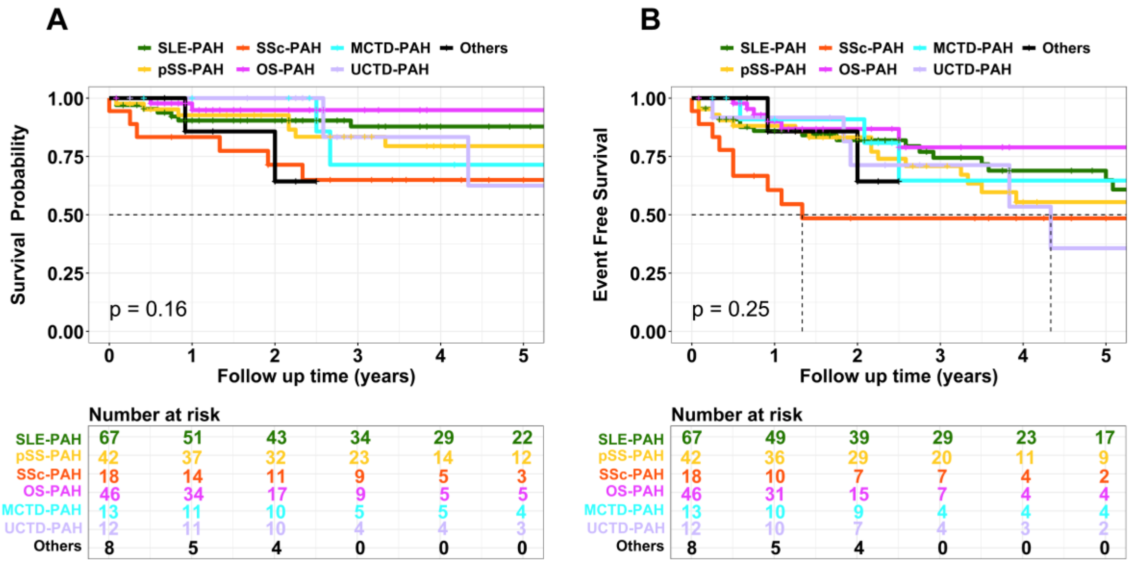

Supplementary Figure 1. Kaplan–Meier curves by CTD subtype for (A) overall survival and (B) event-free survival (clinical failure = all-cause mortality or hospitalization for worsening PAH). Median follow-up: 34.000 (27.756–40.244) months. Events by subtype — Deaths (*n* = 28): SLE 7, pSS 7, SSc 6, OS 2, MCTD 2, UCTD 2, other 2. Clinical failure events (*n* = 57): SLE 17, pSS 15, SSc 9, OS 6, MCTD 3, UCTD 5, other 2. Numbers-at-risk are displayed below each plot.

Supplementary Table 1. Diagnostic information for overlap syndromes (OS)

**OS**	***n* = 46**
SLE AIH, *n*	1
SLE RA, *n*	1
SLE SS, *n*	19
SLE SSc, *n*	2
SLE SS SSc, *n*	4
SLE SS RA, *n*	2
SS PBC, *n*	7
SS AIH, *n*	2
SSc SS, *n*	4
SSc DM, *n*	1
SSc DM RA, *n*	1
SSc SS RA, *n*	1
SSc SS AIH, n	1
